# Commentary: Longitudinal changes in circulating metabolites and lipoproteins after breast cancer treatment

**DOI:** 10.3389/fcvm.2022.962698

**Published:** 2022-08-09

**Authors:** Avirup Guha, Nickolas Stabellini, Alberto J. Montero

**Affiliations:** ^1^Cardio-Oncology Program, Department of Cardiology, Medical College of Georgia at Augusta University, Augusta, GA, United States; ^2^Department of Medicine, Case Western Reserve University School of Medicine, Cleveland, OH, United States; ^3^Graduate Education Office, Case Western Reserve University School of Medicine, Cleveland, OH, United States; ^4^Department of Hematology-Oncology, University Hospitals Seidman Cancer Center, Cleveland, OH, United States; ^5^Faculdade Israelita de Ciências da Saúde Albert Einstein, Hospital Israelita Albert Einstein, São Paulo, Brazil; ^6^Department of Population and Quantitative Health Sciences, Case Western Reserve University School of Medicine, Cleveland, OH, United States

**Keywords:** cardio-oncology, lipid management, cardiometabolic risk, risk factors, hyperlipidemia, breast cancer, treatment

## Introduction

This commentary builds upon the recent paper by Giskeødegård et al. (1) to complement and advance the debate about the cardiovascular (CV) effects of cancer treatment. The aim is to discuss the practical implications of the findings and how this can change cancer care. This can lead to more personalized care and greater integration with cardio-oncology, aiming at cardiovascular safety and preventing adverse cardiac outcomes in cancer patients, delivering value and team-based care.

## Cancer care and its cardiovascular effects

Cancer systemic treatments [e.g., chemotherapy (CT), immunotherapy (IT), radiotherapy (RT), and endocrine therapy (ET)] have emerged as a great weapon against an aggressive and highly prevalent disease, leading to improved quality of life and preventing millions of deaths worldwide. Historically, RT emerged in the late nineteenth century, CT was first used for cancer in the 1930s, and, poly-CT in 1958, while the use of IT began in 1991 with the approval of IL-2 use in metastatic kidney cancer (2, 3). In turn, agents used in ET began to be used in 1978 with the approval of Tamoxifen by the FDA and, later, new classes of drugs, such as aromatase inhibitors (AI) and GnRH analogs/inhibitors (ADT), changed the treatment paradigm of breast and prostate cancers (3).

These critical advances, however, came at a cost: adverse effects, among which one of the main ones are the cardiometabolic (such as hypertension, dyslipidemias, diabetes, and metabolic syndrome) (4–8). mTOR/PI3K-Akt inhibitors (e.g., everolimus and temsirolimus) are associated with hypercholesterolemia [mainly low-density lipoprotein (LDL) and triglycerides (TG)] and hyperglycemia (4). Multi-targeted tyrosine kinase inhibitors are significantly associated with hyperlipidemia and glycometabolic abnormalities, including increased fasting plasma glucose levels (4). Immune checkpoint inhibitors (e.g., ipilimumab, nivolumab, pembrolizumab, atezolizumab, avelumab, and durvalumab) can cause accelerated atherosclerosis, hyperglycemia, and type 1 diabetes mellitus (DM-1) (4). Among the drugs used for endocrine therapy, AIs are shown to increase the risk of dyslipidemia, hyperglycemia, metabolic syndrome and hypertension, while ADTs are associated with hypertension, hyperglycemia and metabolic syndrome (4). In addition to these, other classes related to the development of dyslipidemias are anthracyclines, VEGH inhibitors, L-asparginase, JAK 1/2 inhibitor, Bexarotene, and Capcitabine (5).

In this knowledge base, the study by Giskeødegård et al. makes significant additions for patients treated for breast cancer. With a prospective design, the authors recruited 250 breast cancer patients referred for post-operative local or locoregional RT between 2007 and 2008. These patients were treated following the recommendations of the Norwegian guidelines (including CT, ET, IT, in addition to surgery and RT), separated into groups according to treatment, and had serum samples collected at 5 time points (before of RT, after the end of RT, and 3, 6, and 12 months after RT, respectively), with measurement of lipoprotein parameters. The results showed the development of an atherogenic profile (especially in TG, which agrees with reports in the literature that point to increases of up to 100%, mainly in the use of mTOR inhibitors) independent of treatment type, with a decrease in esterified cholesterol and an increase in free cholesterol of all high-density lipoprotein (HDL) subfractions and large LDL particles (9). Interestingly, considering a 10-year follow-up, the authors also demonstrate that non-survivors had lower cholesterol levels than survivors in the pre-RT period.

The novelty brought by the study is the demonstration of this increase stratified by subfractions, in different groups and different time periods, pointing to the need for a careful and accurate assessment of patients and the treatment used to seek not only the tumor control but also the prevention of undesirable events, such as cardiovascular events. Several medical societies and specialists have CV assessment recommendations for patients before and during treatment, and these findings add data and knowledge to support additions on the recommendations (10–18).

## The role of cardio-oncology

**Figure 1:**
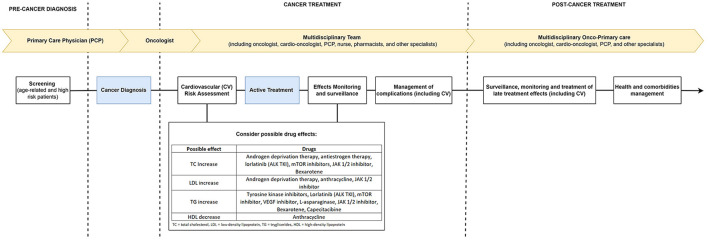
Suggested care framework for cancer patients with multidisciplinary teams.

Cardio-oncology is responsible for the cardiovascular care of cancer patients and works based on the undesired cardiovascular mechanisms and effects of cancer therapies (19, 20). Its performance is based on developing risk strategies (either primary or secondary) for prevention and intervention aimed at reducing CV risk, preventing cardiotoxicity, and managing adverse effects (20, 21). Epidemiological trends demonstrate the apparent association between cancer, its therapies, and CV events, whether they are outcomes (as proven in other studies) or profile changes [as demonstrated by Giskeødegård et al. (1)]. This work is yet another demonstration of the critical role and the need for this specialty to be increasingly integrated into the care of cancer patients, acting through proposals and interdisciplinary teams to make the care of these patients as qualified as possible (Figure 1).

Combining the results of previous work with the novelties brought by this study, we can conclude the need to not only closely monitor the metabolic profiles of patients, but also to understand the impact of each drug and each type of treatment and period of time on the subfractions of each component of the cardiometabolic system in order to act in a targeted manner always keeping in mind the dyslipidemic potential of the treatment in general (22, 23). A very clear example is triglycerides, which showed a significant increase in this and other studies, demonstrating that this lipid subfraction deserves extreme attention in prescribing treatment and in the follow-up of patients over time. In addition, as pointed out by the authors, confounding factors, such as lifestyle, are contributors to this lipoprotein change and should also be the target of action by the medical team before considering drug prophylaxis (such as statins) (24).

## Future directions

The findings reported by the mentioned study should be interpreted considering that the authors selected a convenience sample containing only patients referred for post-operative RT, a treatment that defined the time-points. Although this treatment is highly prevalent (according to the National Cancer Institute, 52.6% of breast cancer patients are treated with surgery followed by RT), the findings should not be extrapolated to other populations, as radiotherapy may also have had an effect (25). The definition of time-points allowed a standardization method, however, differences in time-to-treatment may also have affected the results and not completely reflect the effect of each type of treatment.

Therefore, this study and its findings present important novelties for the field that may culminate in additions to the current guidelines, however other studies needs to consider patients who do not undergo RT and to establish time-points according to each type of treatment for a better understanding of this topic.

## Author contributions

AG and NS drafted the first version of the manuscript. All authors contributed to the article and approved the submitted version.

## Funding

AG was supported by American Heart Association-Strategically Focused Research Network Grant in Disparities in Cardio-Oncology (#847740 and #863620). NS was supported through funding from the Sociedade Beneficente Israelita Brasileira Albert Einstein on the program-Marcos Lottenberg & Marcos Wolosker International Fellowship for Physicians Scientist - Case Western.

## Conflict of interest

The authors declare that the research was conducted in the absence of any commercial or financial relationships that could be construed as a potential conflict of interest.

## Publisher's note

All claims expressed in this article are solely those of the authors and do not necessarily represent those of their affiliated organizations, or those of the publisher, the editors and the reviewers. Any product that may be evaluated in this article, or claim that may be made by its manufacturer, is not guaranteed or endorsed by the publisher.
